# Aerosol Oxidative Potential in the Greater Los Angeles Area: Source Apportionment and Associations with Socioeconomic Position

**DOI:** 10.1021/acs.est.2c02788

**Published:** 2022-12-06

**Authors:** Jiaqi Shen, Sina Taghvaee, Chris La, Farzan Oroumiyeh, Jonathan Liu, Michael Jerrett, Scott Weichenthal, Irish Del Rosario, Martin M. Shafer, Beate Ritz, Yifang Zhu, Suzanne E. Paulson

**Affiliations:** † Department of Atmospheric & Oceanic Sciences, University of California, Los Angeles, California 90095, United States; ‡ Department of Environmental Health Sciences, Jonathan and Karin Fielding School of Public Health, University of California, Los Angeles, California 90095, United States; § Department of Epidemiology, Biostatistics, and Occupational Health, McGill University, Montreal, Quebec H3A 1A2, Canada; ∥ Department of Epidemiology, Fielding School of Public Health, University of California, Los Angeles, Los Angeles, California 90095, United States; ⊥ Environmental Chemistry and Technology Program, University of Wisconsin−Madison, Madison, Wisconsin 53706, United States

**Keywords:** reactive oxygen species, brake and tire wear, environmental justice, hydroxyl radical, dithiothreitol, air pollution exposure, PMF, exhaust, nonexhaust, health

## Abstract

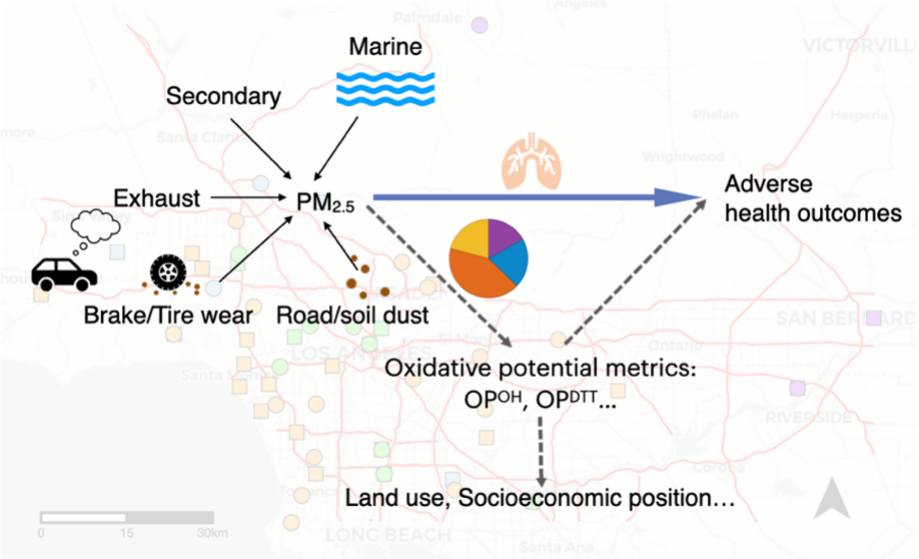

Oxidative potential (OP) has been proposed as a possible integrated metric for particles smaller than 2.5 μm in diameter (PM_2.5_) to evaluate adverse health outcomes associated with particulate air pollution exposure. Here, we investigate how OP depends on sources and chemical composition and how OP varies by land use type and neighborhood socioeconomic position in the Los Angeles area. We measured OH formation (OP^OH^), dithiothreitol loss (OP^DTT^), black carbon, and 52 metals and elements for 54 total PM_2.5_ samples collected in September 2019 and February 2020. The Positive Matrix Factorization source apportionment model identified four sources contributing to volume-normalized OP^OH^: vehicular exhaust, brake and tire wear, soil and road dust, and mixed secondary and marine. Exhaust emissions contributed 42% of OP^OH^, followed by 21% from brake and tire wear. Similar results were observed for the OP^DTT^ source apportionment. Furthermore, by linking measured PM_2.5_ and OP with census tract level socioeconomic and health outcome data provided by CalEnviroScreen, we found that the most disadvantaged neighborhoods were exposed to both the most toxic particles and the highest particle concentrations. OP^OH^ exhibited the largest inverse social gradients, followed by OP^DTT^ and PM_2.5_ mass. Finally, OP^OH^ was the metric most strongly correlated with adverse health outcome indicators.

## Abstract 2

We measured PM_2.5_ oxidative potential with the hydroxyl radical and dithiothreitol assays and investigated its sources to associations with land use type, neighborhood socioeconomic position, and adverse health outcomes.

## 1 Introduction

Airborne particulate matter (PM) smaller than 2.5 μm in diameter (PM_2.5_) is widely recognized as contributing to an extensive range of adverse health outcomes, including all-cause mortality, cardiovascular mortality, cardio-respiratory morbidity, metabolic diseases such as diabetes, cognitive decline, neurological disorders, and adverse birth outcomes.^1,2^ A leading hypothesis of why PM might be responsible for some of these adverse health effects is the induction of oxidative stress, an imbalance between reactive oxygen species (ROS) and antioxidant defenses in the cell.^3^ Inhaled PM can contribute to excess ROS via both particle-bound ROS and ROS generated by the interaction of PM components with antioxidants, proteins, and other species.^4^

A range of acellular oxidative potential (OP) assays have been developed as a possible metric for evaluating particle-induced adverse health outcomes complementary to PM mass.^4−6^ OP assays can be divided into assays that measure oxidant production and those that measure the depletion of common lung antioxidants or other organic reductants. Oxidant production assays include the hydroxyl radical (OH) assay and the electron paramagnetic resonance (EPR) assay.^7,8^ The OH assay measures the formation of the most reactive ROS species (OH) in surrogate lung fluid containing the major lung antioxidants, and the EPR assay measures particle-bound free radicals. Depletion assays include the ascorbic acid (AA) assay, glutathione (GSH) assay, and, indirectly, the dithiothreitol (DTT) assay, an assay carried out in phosphate buffer.^9−11^ While both AA and GSH are important cellular and extracellular antioxidants, DTT is viewed as a surrogate for biological reductants.

Trace metals are important drivers of OP responses for both the OH and DTT assay. The two assays, however, respond differently to different metals. For example, the OH assay is fairly sensitive to Fe, while the DTT assay is much less affected by Fe, making the DTT assay less representative in capturing the ROS generated through Fenton chemistry or synergistic effects.^5^ In addition to metals, both assays appear to be sensitive to specific organics, either directly or via interactions between metals and organics. DTT activity has been found to be associated with organic carbon, quinones, humic-like substances (HULIS), secondary organic carbon, and biomass-burning organic aerosols.^5,12^ A very limited number of studies have also shown the association between OH assay and organics,^13,14^ although more investigation is needed. Many questions remain regarding the OP assays, including which assays are most strongly related to health outcomes and which components in particles produce the signals observed in the assays.

Numerous regulations aimed at tailpipe emissions have substantially reduced pollution from this source, even after accounting for the large increases in vehicle miles traveled (VMT) over the past decades.^15^ In the absence of similar regulations for non-tailpipe particles, the relative proportion of these particles as part of total on-road emissions has increased.^16^ Increasing VMT has also increased the absolute emissions from these sources. Thus, simultaneous decreases in elemental carbon and polycyclic aromatic hydrocarbons (PAHs) and increasing concentrations of metals in PM_2.5_, many associated with brake and tire wear and road dust, have been observed over time.^17,18^ Generally consistent with the increase in redox-active metals, Shirmohammadi, et al.^18^ reported a small increase in the mass-normalized aerosol oxidative potential measured by the DTT assay for PM_2.5_ collected in the Los Angeles area between 2002 and 2013.

People in lower socioeconomic position (SEP) often face double jeopardy, whereby they have worse environmental exposures and heightened susceptibility to those exposures due to higher rates of preexisting conditions and less access to medical care and healthy foods leading to poor nutrition that puts them at higher risk.^19^ Thus, individuals and neighborhoods with lower SEP are likely to suffer worse health impacts from pollutant exposure than those in higher socioeconomic position.^20−22^ Studies have found that lower SEP neighborhoods in Los Angeles were exposed to higher levels of PM_2.5_ mass and nitrogen dioxide;^21,23^ however, less common are studies that describe variations in intrinsic toxicity of air pollution in neighborhoods according to relative SEP.

This study was performed to (1) assess the relationships between the OPs and PM_2.5_ mass, BC, and elements from both volume-normalized (i.e., measured value per m^3^ of air) and mass-normalized (i.e., measured value per μg of particulate matter) perspectives; (2) determine the role of vehicular nonexhaust emissions to OP for the Greater Los Angeles area using PMF, given its increasing contribution to PM mass concentrations; (3) explore how PM_2.5_ mass, OP^OH^, and OP^DTT^ vary by neighborhood SEP using the CalEnviroScreen database; and (4) investigate the relationship of PM_2.5_ mass and OP with various health endpoints in CalEnviroScreen, with the hope of adding more evidence to the limited database of studies relating acellular OP assays to health outcomes.

## 2 Methods

### 2.1 Sample Collection

Ambient PM_2.5_ samples were collected across the Greater Los Angeles, California, area during September 2019 and February 2020. For each season, 27 samples (54 in total) were collected in parallel over a two-week period at different sites. Four sites were repeated in both seasons for 50 total sampling locations, which included background, desert, community, and traffic sites (Figure S1; see Oroumiyeh et al.^24^ for a detailed description of the site classification criteria).

Particles were collected on precleaned 37 mm Teflon filters (Pall Inc.) with PM_2.5_ impactors (H-PEM, BGI Inc.) at 1.78 ± 0.02 Lpm. Nine and 11 blank filters were collected in summer and winter, respectively, at field sites or in the lab, following the same procedure but with pump on for only 30 s. These blank filters were subjected to the same analyses as samples.

### 2.2 Mass, Black Carbon (BC), and Element Measurements

Filters were weighed using a microbalance (Sartorius ME-5) before and after aerosol sampling in a temperature-, humidity-, and vibration-controlled weighing room. Optical absorption at 880 and 370 nm was measured on the filters prior to chemical analysis using an optical transmissometer (Magee Scientific). A detailed description of the loading and scattering corrections for the BC measurements can be found in SI Section S1.2.

Total concentrations of 52 elements (mainly metals, see Section S1.2 for a list of the elements) were measured for each filter by Sector Field Inductively Coupled Plasma Mass Spectrometry (SF-ICP-MS, Thermo-Finnigan Element 2XR), as described in Oroumiyeh et al.^24^

### 2.3 Aerosol Oxidative Potential Measurements

After measuring PM_2.5_ mass and BC concentration, we halved the filters with ceramic scissors and analyzed one half with the OH assay and the other with the DTT assay. Half filters were wetted using 25 μL of 50% v/v 2,2,2-trifluoroethanol-water and then incubated in surrogate lung fluid (SLF) containing the terephthalate OH probe, and in phosphate buffer containing DTT, for the OH assay and DTT assay, respectively. The phosphate buffer used in the measurements was treated with Chelex 100 Resin (Bio-Rad Laboratories, Inc.) to remove trace metals. OPs measured in this study are mainly responses to soluble PM components but may also include some heterogeneous reactions on the surface of PM.

#### 2.3.1 OH Assay

The OH assay has been described in detail by Gonzalez, et al.^14^ The assay uses terephthalate to measure the OH radical formation in SLF at 37 °C over the course of a 2-hour incubation. Terephthalate reacts with OH to form highly fluorescent 2-hydroxyterephthalate with 33% yield at pH 7.3. 2-Hydroxyterephthalate was quantified at λ_ex_/λ_em_ of 320/420 nm with a fluorescence spectrometer (Scinco, Korea). The volume of the incubation solution was adjusted depending on the total PM mass concentration so that all analyses were performed with a (solution) PM_2.5_ concentration of 25 μg/mL. The SLF used in this study consisted of 200 μM ascorbate, and 100 μM each reduced glutathione and uric acid sodium salt. In earlier studies, we also added citrate,^25^ but we have removed it since we found it not to be physiologically representative.^26^

#### 2.3.2 DTT Assay

The DTT assay measures the decay of 100 μM DTT in phosphate buffer^9^ over a 32-minute incubation period at 37 °C. Samples were incubated at a (solution) PM_2.5_ concentration of 10 μg/mL. DTT was quantified by reacting it with dithiobisnitrobenzoic acid, to form 2-nitro-5-thiobenzoic acid.

A more detailed description of the OH and DTT assays and chemicals used in the assays can be found in the SI (Section S1.3–S1.5).

### 2.4 Data Analysis

The measured OH formation rate, DTT loss rate, and BC concentration data were further converted to mass- or volume-normalized data. Before analyzing the data, element measurements with concentrations below their respective detection limits were replaced by half of the detection limit. Four elements (Pd, Pt, Sc, and Se) had a few values (1, 2, or 3) below the detection limit. Spearman’s correlation analyses were conducted with SPSS software (SPSS Inc., version 27).

We used the US Environmental Protection Agency’s positive matrix factorization (PMF) model version 5.0 to identify major sources and quantify their relative contribution to the volume-normalized OP^OH^ and OP^DTT^ (OP_v_^OH^ and OP_v_^DTT^). OP_v_^OH^ and OP_v_^DTT^ were set to be “total variable” in PMF runs (separately). PMF is a multivariate factor analysis tool that decomposes a matrix of speciated sample data into factor contribution and factor profile matrices by minimizing the objective function (Section S1.6). We included 15 elements including Na, Mg, Al, S, K, Ca, Cr, Mn, Fe, Cu, Zn, Sb, Ba, Pb, and BC for the OH and DTT source apportionment models. None of the elements had any values below their detection limits. The signal-to-noise ratios for the input species were all above 2, and thus they were all categorized as “strong” species. A four-factor solution was chosen as the final solution based on the physical interpretation of the PMF-resolved source profiles, high *R*^2^ values of the measured versus predicted OP_v_^OH^ or OP_v_^DTT^, and built-in PMF uncertainty analyses (i.e., Displacement and Bootstrap). More details regarding PMF model overview, uncertainty calculation, and error estimation criteria are included in Section S1.6.

We further linked PM_2.5_ mass/OP data for each site with the corresponding CalEnviroScreen data for the census tract containing the site. CalEnviroScreen 4.0 (https://oehha.ca.gov/calenviroscreen/report/calenviroscreen-40) is the latest iteration of the California Communities Environmental Health Screening Tool released in 2021 by the California Office of Environmental Health Hazard Assessment (Sacramento, CA). The database consists of quantitative metrics describing pollution exposure (see the SI), health outcomes, and SEP (see the Results and Discussion section) for census tracts, which generally include 3000–7000 people. To compare PM_2.5_ mass concentration and aerosol oxidative potential to socioeconomic position and other factors, we first removed the seasonal influence on PM_2.5_ mass and OP data by subtracting the seasonal average of each metric from the corresponding value for each sample and dividing it by the corresponding seasonal standard deviation. We then linked the deseasonalized PM_2.5_ and OP data with CalEnviroScreen by assigning the PM_2.5_/OP data to the 51 census tracts (one site was at the border of two tracts), in which our monitors were placed and identifying the CalEnviroScreen indicators (six exposure indicators, five socioeconomic factor indicators, and three health outcomes indicators). The percentile rankings of these indicators were used for correlation analysis.

## 3 Results and Discussion

### 3.1 Seasonal Variability and Relationships between PM_2.5_ Mass Concentration, OP^OH^, and OP^DTT^

Table 1 summarizes the statistical characteristics of volume- and mass-normalized OP^OH^ and OP^DTT^ (OP_v_^OH^, OP_m_^OH^, OP_v_^DTT^, and OP_m_^DTT^) during summer and winter as well as PM_2.5_ mass, BC Fe, Cu and Mn concentrations. PM_2.5_ mass concentration, OP_v_^OH^, and OP_v_^DTT^ were higher in winter than in summer by 16, 54, and 53%, respectively; wintertime OP_m_^OH^ and OP_m_^DTT^ were 31 and 32% higher, respectively. Winter is characterized by less photochemically generated secondary aerosol formation, more partitioning of semi-volatile organic compounds into the particles, and lower vertical mixing heights, resulting in somewhat higher PM_2.5_ mass concentrations and lower contributions from some secondary organics and inorganic ions.^27^ As expected, given the higher mass concentrations in winter, volume-normalized OP (OP_v_^OH^ and OP_v_^DTT^) was higher. Even after controlling for mass, however, OP (OP_m_^OH^ and OP_m_^DTT^) was still higher in the winter. Consistent with this, higher mass fractions of key metals such as Cu, Fe, and Mn were observed in winter (Table 1). For a detailed discussion of the comparison of the metals, and PM_2.5_ mass concentrations with earlier studies, see Oroumiyeh et al.^24^

**Table 1:** Average Concentrations, Standard Deviations, and Mass-Normalized Values for PM Mass, OP, and Selected Metals for Both Seasons

	PM_2.5_ (μg/m^3^)	OP_v_^OH^ (pmol/min/m^3^)	OP_v_^DTT^ (pmol/min/m^3^)	BC (μg/m^3^)	Cu (ng/m^3^)	Fe (ng/m^3^)	Mn (ng/m^3^)
summer	8.0 ± 1.5	3.9 ± 1.3	430 ± 100	0.32 ± 0.11	7 ± 5	150 ± 90	2.8 ± 1.7
winter	9.3 ± 2.5	6.0 ± 2.2	660 ± 220	0.50 ± 0.18	11 ± 6	230 ± 100	3.9 ± 1.5
		OP_m_^OH^ (pmol/min/μg)	OP_m_^DTT^ (pmol/min/μg)	BC (mg/g)	Cu (mg/g)	Fe (mg/g)	Mn (mg/g)
summer		0.48 ± 0.10	53 ± 5	39 ± 9	0.75 ± 0.4	16 ± 7	0.29 ± 0.12
winter		0.63 ± 0.13	70 ± 10	52 ± 12	1.0 ± 0.4	23 ± 6	0.39 ± 0.09

The intrinsic OH activity (OP_m_^OH^) measured in this study (averages of 0.48 and 0.63 pmol min^–1^ μg^–1^ for summer and winter, respectively) was a bit higher than 0.3 pmol min^–1^ μg^–1^ previously measured in Los Angeles in late summertime 2014 at a single site impacted either by air masses with urban aerosols containing relatively high amounts of secondary organic aerosol or by air masses from an unpopulated mountain area, depending on time of day.^28^ Our data was in the range of values measured by Li et al.^13^ for an urban and a suburban site in China (0.2–1.2 pmol min^–1^ μg^–1^) in summer 2014. The intrinsic DTT activity (OP_m_^DTT^) observed here (averaging 53 and 70 pmol min^–1^ μg^–1^ for summer and winter, respectively) falls within the range of intrinsic DTT activity measured from traffic emissions in other locations in the United States.^5^ Several earlier studies in Los Angeles, however, reported lower OP_m_^DTT^, at around 15–30 pmol min^–1^ μg^–1^, and also lower OP_v_^DTT^ (100–400 pmol min^–1^ m^–3^) than we observed (340–750 pmol min^–1^ m^–3^, Table 1).^18,29^ A potential explanation for this discrepancy may be that the earlier studies did not control for the mass concentration of particles in the DTT solutions; Charrier et al.^30^ showed that the DTT response per unit mass of particles can decrease by a factor of three over the range 5–40 μg/mL, the concentration range used in the earlier studies. We used a constant value at the lower end of this range (10 μg/mL); thus, higher values might be expected.

**Figure 1:**
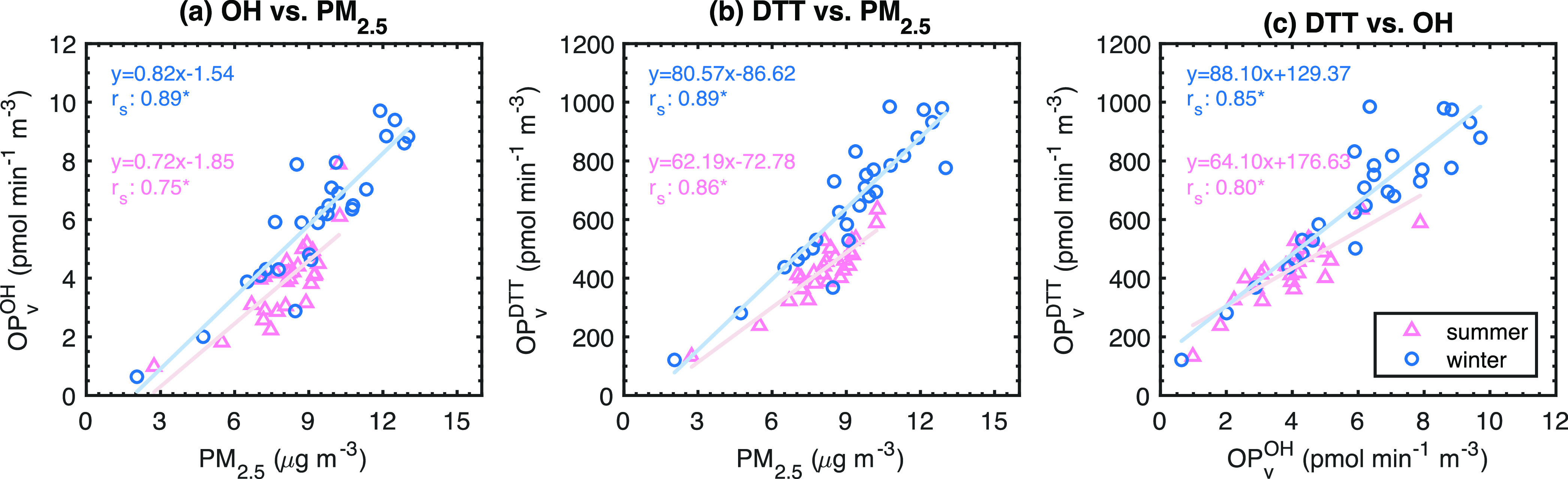
Regression analysis between PM_2.5_ mass concentration and OP_v_^OH^ and OP_v_^DTT^. * indicates *p* < 0.05.

Spearman’s correlations (*r*_s_) between PM_2.5_ mass and OP_v_^OH^ and OP_v_^DTT^ are shown in Figure 1. Both OP_v_^OH^ and OP_v_^DTT^ are strongly correlated with PM_2.5_ mass, but the OP_v_^DTT^ (*r*_s_ = 0.86–0.89) correlation is somewhat stronger than OP_v_^OH^ (*r*_s_ = 0.75–0.89); correlations are slightly stronger in winter. In addition, OP_v_^OH^ and OP_v_^DTT^ strongly correlate with each other (*r*_s_ = 0.80–0.85).

SI Figure S2 shows correlations of OP_m_^OH^, OP_m_^DTT^, and PM_2.5_ mass. OP_m_^OH^ and OP_m_^DTT^ were less correlated with each other (*r*_s_ = 0.53–0.56, *p* < 0.05) than OP_v_^OH^ and OP_v_^DTT^. OP_m_^DTT^ was not statistically significantly correlated with PM_2.5_ mass in summer at *p* < 0.05 and was moderately correlated in winter (*r*_s_ = 0.53). OP_m_^OH^ had a moderately sized positive association with PM_2.5_ mass concentration in both seasons (*r*_s_ = 0.42 and 0.54, *p* < 0.05 for summer and winter, respectively). The observed trend contrasts with many other studies,^13,31,32^ in which an inverse relationship between PM_2.5_ mass and mass-normalized OP was observed, a phenomenon that has been attributed to OP-inactive or low-active components such as inorganic ions that added to the PM mass on highly polluted days.^13^ In contrast to our study, for which 2-week average PM_2.5_ mass concentrations were below 13 μg m^–3^, the mass concentration in these studies reached 100 μg m^–3^ or even higher. Additionally, the mass fractions (or mass-normalized concentrations) of Fe and Cu were positively correlated with PM_2.5_ mass. Our observed positive correlation between OP_m_^OH^, OP_m_^DTT^, Fe, and Cu with PM_2.5_ may also reflect an increasing contribution of urban particles as the PM_2.5_ mass concentration increases. Urban particles are expected to have higher concentrations of available metals (at least partly due to higher solubility) and possibly more active organics relative to background/non-anthropogenic marine aerosols.

### 3.2 Correlations between OP, BC, and Elements

Oxidative potential (OP_m_^OH^ and OP_m_^DTT^) exhibited moderate to strong correlations with tracers of exhaust emissions, including BC, Rh, Pt, and Pd, brake and tire wear tracers such as Ba, Cu, and Sb, and metals associated with industry such as Ag and Cd, while there were overall negative correlations between OP and marine tracers such as Na, Mg, and V (SI Section S3).

### 3.3 OP Source Apportionment

#### 3.3.1 OP_v_^OH^ Factor Identification

**Figure 2:**
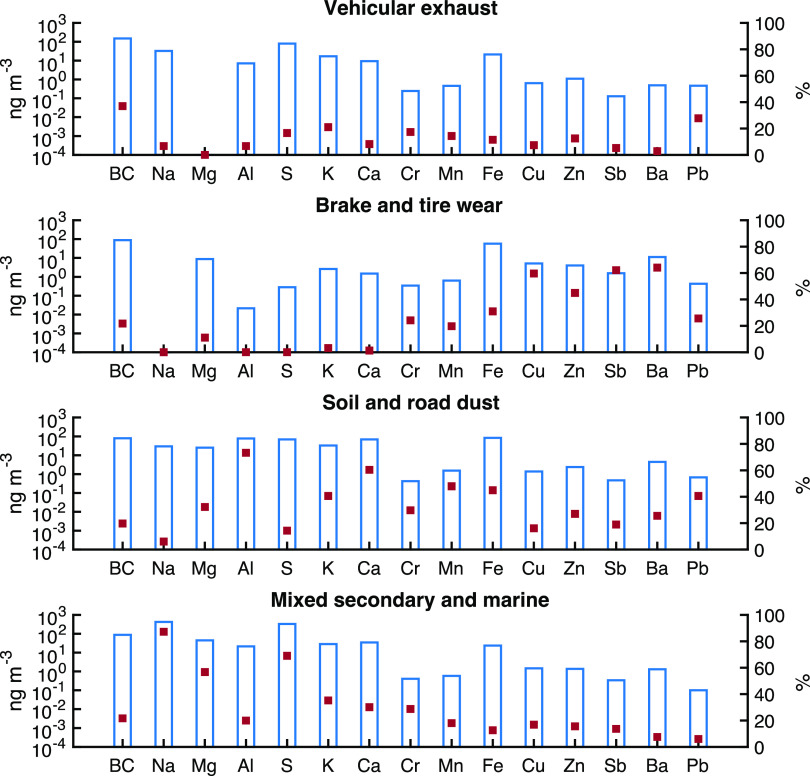
Factor profiles of the OP_v_^OH^ PMF model. The bars (left axis) represent the concentration of species for each factor on a log scale, and the dots (right axis) denote the percentage contribution of each factor to the total concentration of each species.

**Figure 3:**
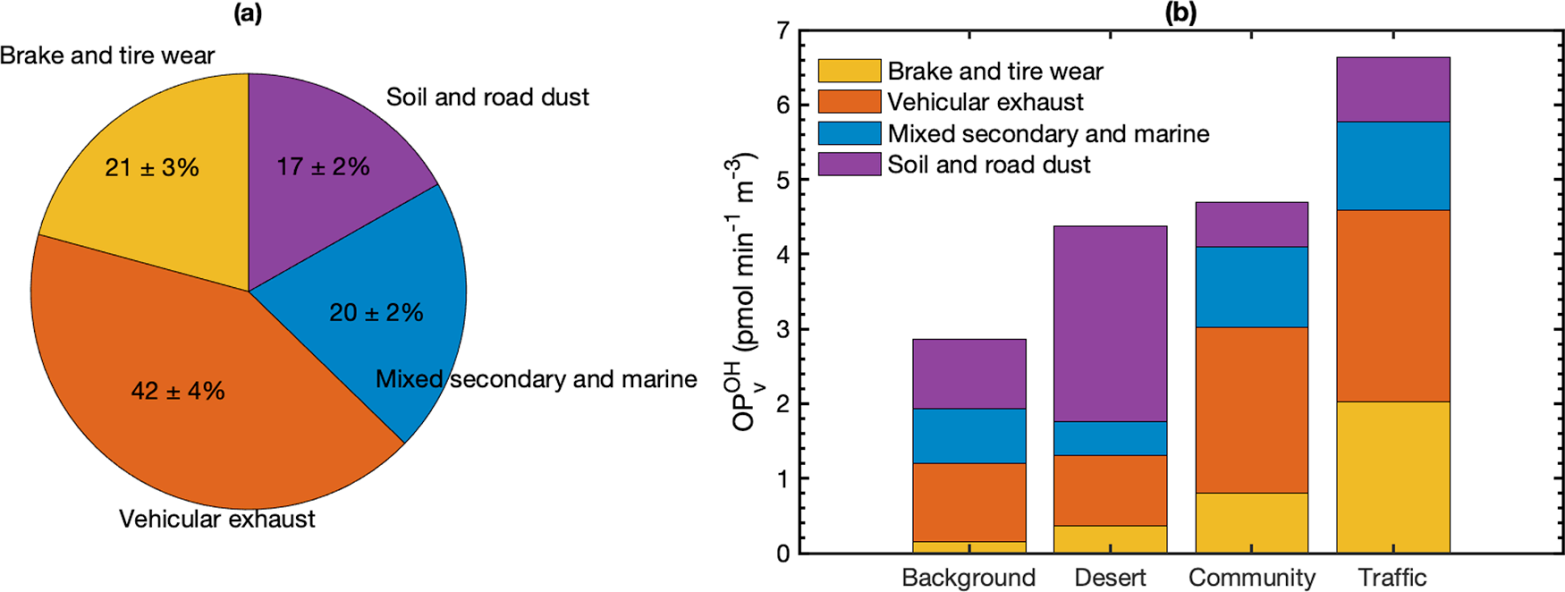
(a) The average contribution of PMF-resolved sources to OP_v_^OH^ for all sites (both seasons included) with standard error of the mean and (b) a descriptive comparison of contributions to each site category.

The source apportionment model identified four sources contributing to OP_v_^OH^ (Figure 2), with an excellent R^2^ of 0.92 and slope of 0.95 between the predicted and measured OP_v_^OH^ (SI Figure S4), indicating that the PMF model was able to predict OP_v_^OH^ quite well. Factor 1, characterized by high loadings of BC and Pb (37 and 28%, respectively), with some K, Cr, sulfur, and Mn present in this factor as well, represents exhaust emissions. Previous studies have documented that BC is a major chemical tracer for tailpipe emissions.^33^ The global phaseout of Pb in gasoline at the end of the last century has drastically reduced airborne Pb concentrations. However, Pb is a geogenic impurity in crude oil, so gasoline still contains some Pb; higher amounts have been associated with diesel fuel and motor oil.^34^ Pb has been associated with vehicular emission in studies in both China and Europe.^35,36^ Overall, dust containing historical Pb and resuspended by traffic may be the dominant source of airborne Pb, but much of this lead appears in the coarse size fraction. For the PM_2.5_ fraction studied here, the dust component is not expected to be as dominant.^37^ The presence of K in this factor can be attributed to the use of K in unleaded fuels and some types of oils.^38^ Fossil fuel combustion is also commonly associated with SO_2_ emissions, and Ti and Cr can be emitted by diesel vehicles.^39^ Overall, vehicular exhaust emissions are the largest contributor to OP_v_^OH^, with a contribution of 42% (Figure 3(a)).

Factor 2 is dominated by high factor loadings for Ba, Sb, Cu, and Zn (Figure 2), representing a brake and tire wear source. Previous studies have indicated that Cu is a high-temperature lubricant commonly used in brake pads.^40,41^ Sb is another typical lubricant used in the brake lining to reduce vibrations and improve friction stability.^42^ Ba is also used as a filler in brake pads,^40^ and Zn is believed to originate largely from brake and tire wear and engine lubrication oil.^43^ While Fe is found to have multiple anthropogenic and geogenic sources, it is more related to brake and engine wear.^44^ Additionally, the PMF attributed 20–30% of Pb, Cr, and Mn to this factor profile (Figure 2); these are also associated with brake wear.^45^ This factor accounts for 21% of OP_v_^OH^ (Figure 3(a)).

Factor 3 represents a mixture of soil and road dust. It is characterized by high loadings of crustal elements such as Al, Ca, Mn, and Fe, all of which are well-known tracers of soil^46,47^ and K, which also has a crustal origin.^48^ Further, anthropogenic metals such as Pb, Cr, Zn, Ba, Sb, and Cu loading on this factor are related to brake and tire wear, indicating this factor is not purely soil dust; instead, it also contains contributions from resuspended road dust. Factor 3 contributes 17% to OP_v_^OH^.

The last PMF-resolved factor attributed to a mixture of secondary and marine sources was dominated by Na and Mg, major components of sea salt, as well as S, which is a tracer for secondary aerosols.^49,50^ The presence of BC in this marine factor profile can be attributed to emissions from the very active ports of Los Angeles and Long Beach.^51^ We also observed a moderate amount of Ca and K, constituents of seawater.^52^ Aged sea salt may also contribute to the S in this factor.^53^ Sodium chloride in fresh sea salt can be transformed into sodium sulfate by sulfur dioxide in the atmosphere,^53^ the latter is emitted by fuel oils used in the ships at the ports of Los Angeles and Long Beach as well as stationery and area sources.^54−57^ Overall, factor 4 contributes 20% to OP_v_^OH^.

Because OP_v_^OH^ is a combination of the mass concentration of particles and their intrinsic activity in the OH assay, ideally, we would be able to disentangle the two contributions. Unfortunately, a reliable PMF analysis of PM_2.5_ mass was not possible because we had measurements of only a minority of the components of PM_2.5_ composition; BC and elements measured here contributed only about 24 ± 6% of the PM_2.5_ mass.

#### 3.3.2 Spatial Pattern of OP_v_^OH^ PMF Results

To further probe the validity of our source apportionment results and explore the spatial variability of OP_v_^OH^ sources, Figure 3b shows the mean source contributions to OP_v_^OH^ for four types of study sites: background, desert, community, and traffic.^24^ The site categories have large differences in OP_v_^OH^, differing by a factor of 2.3 between the traffic and background sites, with the desert and community sites in between. Overall, the contributions of PMF-resolved sources to OP_v_^OH^ are consistent with expectations for each site category. Vehicular exhaust emissions constituted a major fraction of OP_v_^OH^ in all sites, but the most to traffic sites followed by the community sites. Brake and tire wear contributed heavily to the OP_v_^OH^ in traffic sites, followed by community sites, and its contribution to background sites was minimal. The mechanically generated brake and tire wear particles are expected to be at the upper end of the PM_2.5_ size range with relatively high densities and are consequently expected not to travel as far as the smaller tailpipe particles. The desert sites in this study were located on the east and north edges of the Los Angeles Basin and are therefore the farthest from the Pacific Ocean, and the secondary and marine sources contributed little at the desert sites. At the desert sites, we see the largest contributions in both absolute and fractional terms from soil. While many of the pairwise differences in contributions shown in Figure 3(b) are statistically significant, some are not; thus, this figure should be considered qualitative.

#### 3.3.3 Source Apportionment for OP_v_^DTT^

Similar factor profiles were observed for OP_v_^DTT^ (SI Figure S5), indicating vehicular exhaust and road dust, mixed secondary and marine, soil, and brake and tire wear as major contributors to OP_v_^DTT^. The main difference for OP_v_^DTT^ is that vehicular exhaust emissions is mixed with road dust in the PMF factor profiles. This mixture has been observed in previous source apportionment analyses as well.^58,59^ Vehicular exhaust mixed with road dust is still the largest contributor to OP_v_^DTT^, with a contribution of 42%, followed by 15–23% each for the other three sources (SI Figure S6a); the spatial pattern of the OP_v_^DTT^ sources is also similar to that for OP_v_^OH^, as shown in SI Figure S6b. The *R*^2^ between predicted and measured OP_v_^DTT^ was 0.78, with a slope of 0.93 (Figure S7).

#### 3.3.4 Source Apportionment Limitations

We did not have tracers for biomass burning such as levoglucosan and K^+^/K.^60^ But with K included in the PMF model as a potential tracer,^61^ we did not identify a biomass-burning source. The lack of a biomass burning source is consistent with the observed average Ångström exponent of about 0.8 (see Supplement S1.2 for an explanation). Los Angeles is at times impacted by wildfires, but wildfires were absent during our sampling periods. Residential wood burning is much less common in Los Angeles than in many urban areas. The dominant role of fossil fuel combustion in total BC in the Los Angeles area is consistent with earlier studies.^62,63^

Specific organics clearly play a role in the OP assays, both directly and by modifying the redox activities of metals through complexation. The contribution of metals, organics, and their interactions with the OPs for different types of aerosols, however, still remain a puzzle. Organic carbon data would clearly be preferable.

Our final OP_v_^OH^ and OP_v_^DTT^ PMF solutions had acceptable statistical characteristics (see SI Section S4). However, a larger number of samples than the number used here (54) might have reduced uncertainties and increased the statistical power of the PMF model.^65−67^ Further, a more comprehensive measurement of the particles, such as one including both water-soluble metals and organics, might also have improved source apportionment for the OH and DTT assays.

### 3.4 Oxidative Potential, Socioeconomic Position, and Health Outcomes

#### 3.4.1 PM_2.5_/Oxidative Potential and Socioeconomic Factors

Table S1 shows Spearman’s correlations for PM_2.5_ and OP and five socioeconomic factors: educational attainment, housing-burdened low-income households, linguistic isolation, poverty, and unemployment. Socioeconomic factors showed weak to strong correlations with each other. Most socioeconomic factors were either weakly or moderately correlated with PM_2.5_ mass, volume- and mass-normalized OP (*r*_s_ = 0.30–0.55). OP_v_^OH^ and OP_m_^OH^ had similar correlations with socioeconomic factors, while OP_m_^DTT^ was more weakly correlated with socioeconomic factors compared with OP_v_^DTT^.

**Figure 4:**
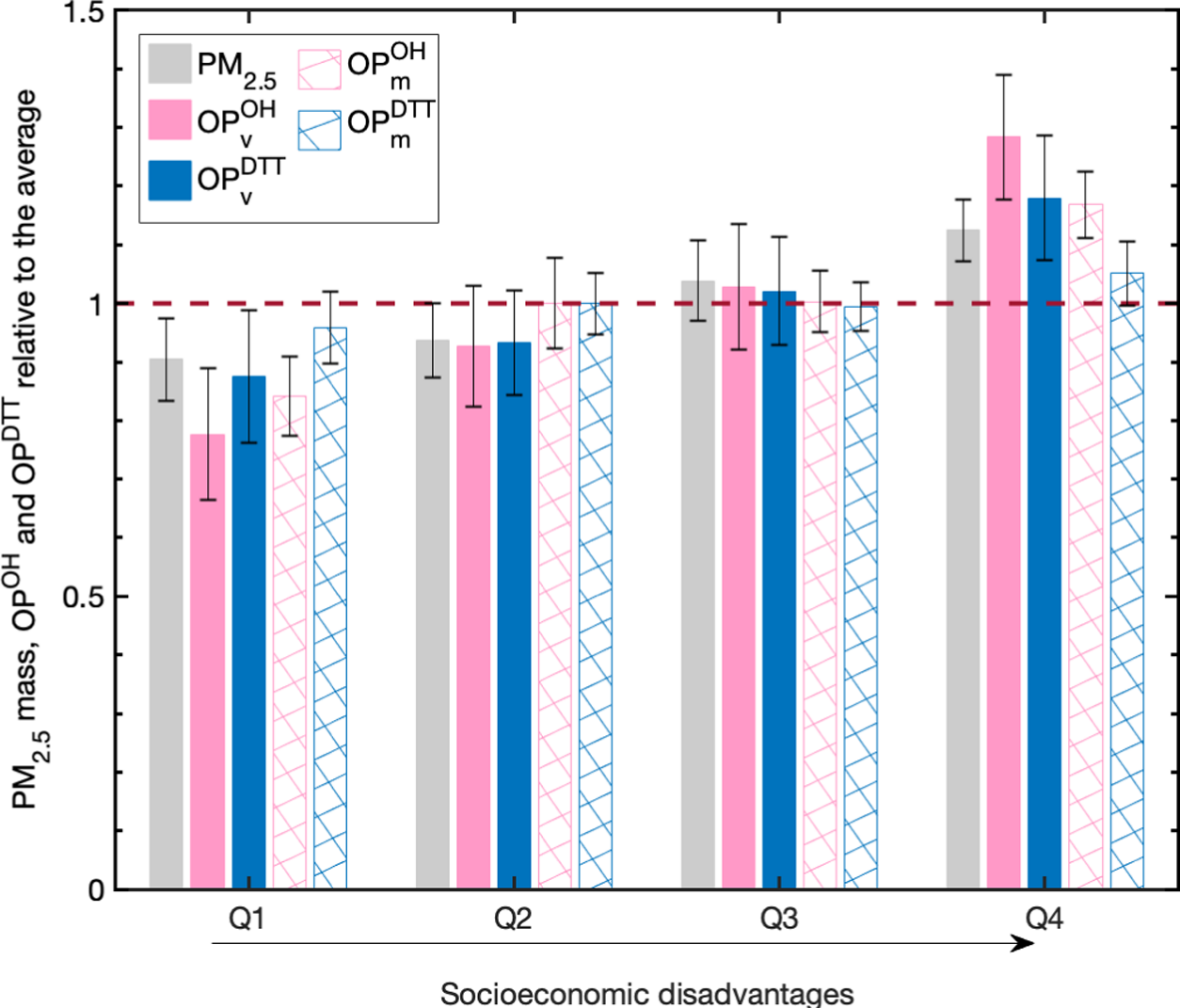
Average PM_2.5_ mass and oxidative potential for each quartile of socioeconomic classification, with data in both seasons included. Error bars represent the standard error of the mean. The dashed line indicates the average of all sampling sites for each metric.

To further explore relative particle toxicity experienced by neighborhoods with different SEP levels, we divided the sites by SEP quartile based on the grouped socioeconomic factors defined in CalEnviroScreen. The number of summer and winter sampling locations was nearly equal for each group. Therefore, the average PM_2.5_ mass, OP_v_^OH^, OP_v_^DTT^, OP_m_^OH^, and OP_m_^DTT^ for each socioeconomic group quartile are plotted in Figure 4. PM_2.5_, OP_v_^OH^, and OP_v_^DTT^ levels consistently increase with increasing socioeconomic disadvantage. People in the most disadvantaged census tract SEP quartile experienced the highest levels of pollution. On average, the most disadvantaged group was exposed to 24, 65, 35, 39, and 10% more PM_2.5_ mass, OP_v_^OH^, OP_v_^DTT^, OP_m_^OH^, and OP_m_^DTT^, respectively, compared with people in the highest SEP quartile. The difference in PM_2.5_ mass, OP_v_^OH^, OP_v_^DTT^, and OP_m_^OH^ exposure for the most advantaged and disadvantaged groups was all statistically significant at *p* < 0.05, except for OP_m_^DTT^. Together, this indicates that the higher OP_v_^OH^ level in more disadvantaged neighborhoods was not only the result of higher particle mass concentrations but also because the particles themselves were more toxic. Once normalized to mass (OP_m_^DTT^), the DTT assay, on the other hand, showed little variability across SEP quartiles, revealing that the DTT assay is a more similar metric to PM_2.5_ mass compared with the OH assay for particles in the studied area.

SI Figure S8 shows the contributions of each source type to each census tract socioeconomic quartile. The exhaust factor varied between groups but did not have a clear trend. The contribution of brake and tire wear to OP increased consistently as SEP disadvantages increased, possibly because brake and tire wear particles were larger and thus somewhat more localized. The soil and road dust factor contribution to OP was also higher for lower SEP groups, possibly caused by more re-entrainment of road dust associated with heavier traffic in near-road areas. Mixed secondary and marine emissions also exhibited a pattern of disproportionate distributions, although its contribution to overall differences in OP exposure was relatively small. Figure SI S9 shows BC, Cu, Fe, and Mn by SEP quartile. Of these, Cu varies the most.

#### 3.4.2 PM_2.5_/Oxidative Potential and Adverse Health Outcomes

Table 2 shows Spearman’s correlations between PM_2.5_ mass/OP and three adverse health outcomes included in the CalEnviroScreen (i.e., asthma, cardiovascular disease, and low birth-weight infants) at the census tract level. OP_v_^OH^ and OP_m_^OH^ were significantly correlated with the census tract group prevalence of all three health outcomes (Table 2), with correlations that were weak to moderate (*r*_s_ = 0.33–0.45). PM_2.5_ mass and OP_v_^DTT^ were significantly correlated only with the prevalence of low birth-weight infants (*r*_s_ = 0.38), and OP^DTT^ did not correlate with any health indicators. The low to moderate size correlation coefficients may partly be due to exposure variations within census tracts that this ecologic measure cannot pick up and the lack of coincidence in timing; our exposure data was from 2019 to 2020, while asthma and cardiovascular data were from 2015 to 2017, and the birth outcome data from 2009 to 2015. Consistent with this, the *r*_s_ for our measured PM_2.5_ mass concentration with the CalEnviroScreen PM_2.5_ value was only 0.46 (SI Table S2). Earlier studies found larger associations between OP_m_^DTT^ and asthma and cardiovascular disease compared with PM_2.5_.^68,69^ OP^OH^ has not previously been investigated in an epidemiological context. Our results suggest that the OH assay may be better at predicting particle-induced adverse health outcomes than the DTT assay or PM_2.5_ mass for the aerosol sources present in this study.

**Table 2:** Spearman’s *r* Values for Associations of PM_2.5_ Mass/OP with Adverse Health Outcomes

	this study						CalEnviroScreen health indicators		
		PM_2.5_	OP_v_^OH^	OP_v_^DTT^	OP_m_^OH^	OP_m_^DTT^	asthma	cardiovascular disease	low birth-weight infants
CalEnviroScreen health indicators	asthma	0.17	0.33*	0.18	0.42*	0.14			
	cardiovascular disease	0.23	0.36*	0.21	0.40*	0.15	0.84*		
	low birth-weight infants	0.38*	0.45*	0.38*	0.36*	0.22	0.58*	0.45*	

* Indicates *p* < 0.05. Numbers without asterisks are not statistically significant at *p* < 0.05.

## 4 Implications and Future Outlook

After decades of exhaust control, the share of nonexhaust in overall road traffic emissions has been increasing; EMission FACtors model (EMFAC2021 v1.0.1, California Air Resources Board) estimates that in recent years brake and tire wear emissions have already exceeded exhaust emissions in Los Angeles area. While not an assessment of PM mass, source apportionment analysis in this study, however, identified exhaust emissions as the dominant contributor of oxidative potential, suggesting that the current view of the relative contributions of exhaust and nonexhaust may not be entirely accurate. Many questions remain with regard to the contributions of exhaust and nonexhaust emissions to particle mass, composition, exposure, and health impacts.

Fe and Cu, the two most active metals in the OH assay, are both larger contributors to the brake and tire wear source than the exhaust source. Our measurements, however, were of total metals, not soluble metals, and soluble metals may be different for traffic and brake and tire wear particles. Further, organic chelators, also not characterized here, can dramatically increase or decrease metal activity.^14^ A more comprehensive chemical speciation of aerosol particles would likely be more helpful for the source apportionment analysis.

Our results indicate a disproportionate burden of PM_2.5_ mass and oxidative potential metrics for people living in lower SEP census tracts. Both volume- and mass-normalized OP^OH^ show large inverse gradients with neighborhood SEP, indicating people living in lower SEP census tracts are both exposed to more particle mass and that these particles may be more toxic, a situation that should be explored in more locations with different sources.

Exploratory ecological analysis suggests that OP^OH^ is the measure most strongly associated with CalEnviroScreen health outcome data compared to PM_2.5_ mass and OP^DTT^, suggesting that the OH assay provides a better metric to predict particle-induced adverse health outcomes, although the applicability of this conclusion to other mixtures of aerosol sources needs more study. Differences in the OH and DTT assays are not well understood and may result from both direct differences in how the assays respond to aerosol components and indirect differences resulting from interactions of the antioxidants that are present both in the OH assay and in lung fluid, but not in the solution used for the DTT assay. Both SEP and health indicators in CalEnviroScreen were themselves averaged from different time periods, none of which coincided with our OP data, adding to the uncertainties of the results. Future studies based on contemporaneous data will likely provide a clearer picture of OP, health, and SEP interactions.

## Supporting Information Available

Detailed methods, PM_2.5_ mass and OP relationships, correlations between OP and elements, source apportionment uncertainty analyses and results for OP_v_^DTT^, and associations of OP with socioeconomic position and pollution exposure indicators (PDF)

## Abbreviations Used

BC: black carbon
DTT: dithiothreitol
OP: oxidative potential
OP^OH^: oxidative potential measured by the production of hydroxyl radicals
OP^DTT^: oxidative potential measured by the depletion of dithiothreitol
OP_v_^OH^: volume-normalized OP^OH^
OP_v_^DTT^: volume-normalized OP^DTT^
OP_m_^OH^: mass-normalized OP^OH^
OP_m_^DTT^: mass-normalized OP^DTT^
PM: particulate matter
PMF: positive matrix factorization
SEP: socioeconomic position
SLF: surrogate lung fluid
VWT: vehicle miles traveled
