# Supplementary material for: Aerosol Oxidative Potential in the Greater Los Angeles Area: Source Apportionment and Associations with Socioeconomic Position
Source: Environ Sci Technol. 2022 Dec 6;56(24):17795–804. doi: 10.1021/acs.est.2c02788 (PMC9775201; doi:10.1021/acs.est.2c02788)
Supplement: Supplementary file 1 — es2c02788_si_001.pdf [file es2c02788_si_001.pdf]

Supplementary Information for

Aerosol Oxidative Potential in the Greater Los Angeles Area: Source Apportionment and Associations with Socioeconomic Position

Jiaqi Shen [a], Sina Taghvaee [a], Chris La [a], Farzan Oroumiyeh [b], Jonathan Liu [b], Michael Jerrett [b], Scott Weichenthal [c], Irish Del Rosario [d], Martin M. Shafer [e], Beate Ritz [d], Yifang Zhu [b], and Suzanne E. Paulson [a,]*

a Department of Atmospheric & Oceanic Sciences, University of California, Los Angeles, CA 90095, USA

b Department of Environmental Health Sciences, Jonathan and Karin Fielding School of Public Health, University of California, Los Angeles, CA 90095, USA

c Department of Epidemiology, Biostatistics, and Occupational Health, McGill University, Montreal, Quebec H3A 1A2, Canada

d Department of Epidemiology, Fielding School of Public Health, University of California, Los Angeles, Los Angeles, CA 90095, USA

e Environmental Chemistry and Technology Program, University of Wisconsin–Madison, Madison, WI 53706, USA

This PDF file includes: 18 Total Pages, 9 Figures and 2 Tables.

Sections

Section S1. Methods
- S1.1 Sampling site map
- S1.2 Black carbon (BC) and 52 elements quantification
- S1.3 Chemicals
- S1.4 The hydroxyl radical (OH) assay
- S1.5 The dithiothreitol (DTT) assay
- S1.6 PMF source apportionment analysis

Section S2. Relationships between $PM_{2.5}$ mass concentration, mass-normalized OP^{OH} and OP^{DTT}
Section S3. Correlation between OP and BC, elements
Section S4. Source apportionment results
Section S5. Oxidative potential, pollution burden and socioeconomic position

Figures

Figure S1. Sample site locations and types during two seasons in September 2019 and February 2020 in Greater Los Angeles.
Figure S2. Relationships between OP_m^{OH} and OP_m^{DTT} with $PM_{2.5}$ mass and each other.
Figure S3. Correlation heatmap showing Spearman's r values for mass-normalized OP and selected elements.
Figure S4. PMF predicted vs. measured OP_v^{OH} for 54 samples.
Figure S5. Factor profiles of the OP_v^{DTT} PMF model.
Figure S6. (a) The average contribution of PMF-resolved sources to the OP_v^{DTT} for all sites (both seasons included) with standard error of the mean and (b) for each site category.
Figure S7. PMF predicted vs. measured OP_v^{DTT} for 54 samples.
Figure S8. Contribution of four emission sources to the OP_v^{OH} for different socioeconomic position groups.
Figure S9. BC, Cu, Fe and Mn relative to their average concentration for each quartile of socioeconomic classification.

Tables

Table S1. Spearman's correlations for $PM_{2.5}$ mass, OP and socioeconomic factors.
Table S2. Spearman's correlations between $PM_{2.5}$ mass or OP and exposure indicators from CalEnviroScreen for the 51 census tracts sampled.

References

Section S1. Methods

S1.1 Sampling Site Map

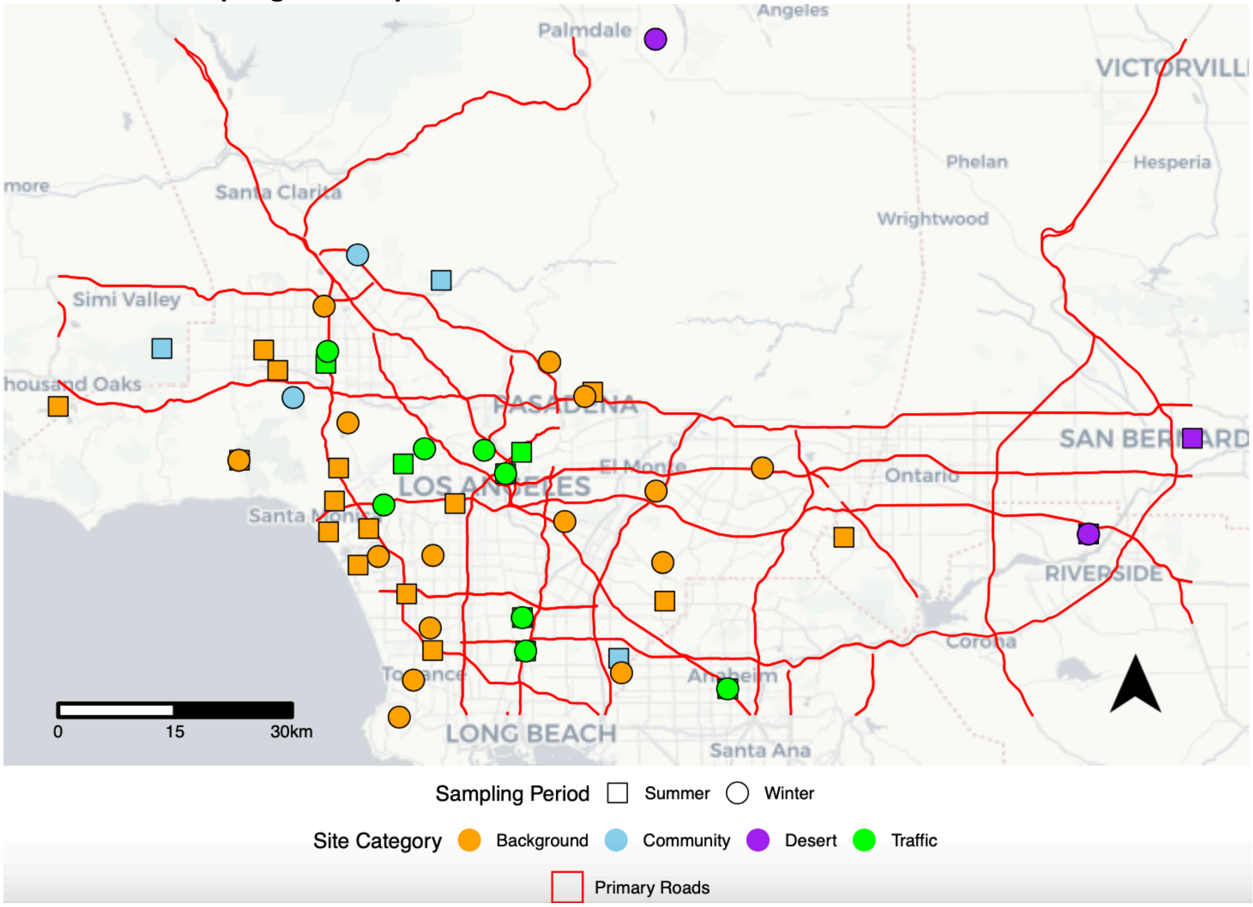

Figure S1. Sample site locations and types during two seasons in September 2019 and February 2020 in Greater Los Angeles. See also Oroumiyeh et al. [1] for a detailed description of the site classifications.

S1.2 Black carbon (BC) and 52 Elements Quantification

We estimated BC on filters from measurements of optical absorption at 370 and 880 nm using an Optical Transmissometer (Magee Scientific). Teflon filters were placed on quartz filters to obtain an even light distribution on the detector. The instrument reports incident and transmitted light, I_0

and I, respectively. For a filter with sampled volume of air, V and filter collection area S, we can calculate the absorption coefficient based on Beer's law using the following equation:

$$b_{ATN} = ln\frac{I_0}{I}\frac{S}{V}. \tag{S1}$$

There are two dominant artifacts associated with filter-based absorption measurements. One is the scattering by the filter fibers, leading to increased light attenuation from multiple opportunities for absorptions, and the other is the shadowing by the particles deposited upon one another, as particles are not perfectly loaded in a single layer. This causes an underestimation of the true attenuation. We corrected the multi-scattering issue and loading effect for the absorption coefficient using the following expression:[2]

$$b_{ATN,corrected} = ln\frac{I_0}{I}\frac{S}{V}\frac{1}{C_{ref}\cdot\{\left(\frac{1}{f_\lambda}-1\right)\cdot\frac{ln\left(In\frac{I_0}{I}\right)-ln(10\%)}{ln(50\%)-ln(10\%)}+1\}}, \tag{S2}$$

where C_{ref} corrects the overestimated attenuation from multiple-scattering and the term in brackets compensates for the loading effect. C_{ref} is a constant for all wavelengths and a value of 2.14 is commonly used for quartz filters.[2-4] The C_{ref} value for PTFE has been reported to be 59% of quartz-fiber filters.[5] Therefore, we used 2.14 × 0.59 as C_{ref} for PTFE filters. The coefficient f_λ is wavelength-dependent, with values of 1.155 and 1.064 for 370 and 880 nm, respectively.[4] Our filters were not heavily loaded; thus the average calculated loading correction factor was only about 1.05 ± 0.02.

Finally, BC concentration is calculated at λ = 880 nm from:

$$BC = \frac{b_{ATN,corrected,\lambda}}{\sigma_\lambda}, \tag{S3}$$

where 16.6 m^2 g^{-1} @ 880 nm was assumed for σ_λ. This value is recommended by the manufacturer for urban traffic-related BC.

The absorption measurements at 880 nm (in the infrared, or IR) only measures black carbon, thus measurements at 880 mm are usually associated with BC from fossil fuel burning.[6] 370 nm (in the ultraviolet, or UV) measures both black and brown carbon; biomass burning aerosol typically contains substantial brown carbon. To have a better idea of the relative abundance of BC from fossil fuel burning vs biomass burning, we also calculated the AAE (Ångström exponent):

$$AAE = \frac{ln\,(b_{ATN,corrected,370}/b_{ATN,corrected,880})}{ln\,(880/370)}. \quad (S4)$$

A high value of AAE indicates the UV absorption is high compared to the IR absorption. AAE = 1 is widely assumed for black carbon but AAE < 1 has been routinely observed in ambient measurements.[7] An AAE value larger than 1 may indicate the presence of significant brown carbon. Total concentrations of fifty-two elements including Li, Na, Mg, Al, P, S, K, Ca, Sc, Ti, V, Cr, Mn, Fe, Co, Ni, Cu, Zn, As, Se, Rb, Sr, Y, Zr, Nb, Mo, Rh, Pd, Ag, Cd, Sn, Sb, Cs, Ba, La, Ce, Pr, Nd, Sm, Eu, Dy, Ho, Yb, Lu, Hf, W, Pt, Hg, Tl, Pb, Th, and U were measured for total concentration using Sector Field Inductively Coupled Plasma Mass Spectrometry (SF-ICP-MS, Thermo-Finnigan Element 2XR).

S1.3 Chemicals

Disodium terephthalate and 2-hydroxyterephthalic acid were purchased from TCI America. Chelex 100 Chelating Resin sodium form (200–400 mesh) was purchased from Bio-Rad. Tris base was purchased from Promega. 10% trichloroacetic acid was purchased from Fisher Scientific. All

other chemicals including ascorbate, reduced glutathione, uric acid sodium salt, DL-dithiothreitol, 5,5-dithiobis (2-nitrobenzoic acid), ethylenediaminetetraacetic acid, sodium phosphate dibasic and potassium phosphate monobasic, copper(II) sulfate pentahydrate and 2,2,2-trifluoroethanol were purchased from Sigma-Aldrich. The highest available purity was selected for all purchases.

S1.4 The Hydroxyl Radical (OH) Assay

The OH assay measures OH radical formation during a 2-hour incubation of samples in surrogate lung fluid (SLF). The SLF consisted of 200 μM Ascorbate, 100 μM each reduced glutathione and uric acid sodium salt dissolved in 10 mM phosphate buffer (114 mM NaCl, 7.8 mM sodium phosphate dibasic and 2.2 mM potassium phosphate monobasic). Solutions were freshly made before each experiment. We incubated the filters in SLF with 10 mM disodium terephthalate (TA) as OH probe at 37 °C in 15 mL Falcon tubes (Corning, Falcon®) and adjusted the volume of SLF for each sample to maintain the $PM_{2.5}$ concentration in the incubation solution at 25 μg/mL to avoid any concentration-dependent effects on the measurements. The product of OH radical and TA (2-hydroxyterephthalic acid, TAOH) was quantified at $\lambda_{ex}/\lambda_{em}$ of 320/420 nm using a Lumina Fluorescence Spectrometer (Thermo Scientific). The yield of TAOH is pH dependent and is 33% at pH 7.3.[8] A calibration curve for TAOH ranging from 0 - 800 nM was constructed daily. The measured OH formation rate for blank filters was about 3 ± 0.5 nM/min. The average blank corrected OH formation rate for samples was about 14 nM/min, an order of magnitude larger than three times of the standard deviation of blanks.

S1.5 The Dithiothreitol (DTT) Assay

For the DTT assay, we based our procedure on Cho et al. [9]. The DTT solution used in the assay consisted of 100 μM DTT in 100 mM phosphate buffer (78 mM sodium phosphate dibasic and 22

mM potassium phosphate monobasic). Half filters were incubated in the 100 μM DTT solution at 37 °C in 50 mL polypropylene centrifuge tubes (Thermo Scientific). $PM_{2.5}$ incubation concentration was also fixed at 10 μg/mL. At 8, 16, 24 and 32 minutes, we took 0.25 mL aliquots of the reaction mixture and added to 0.25 mL of 10% trichloroacetic acid to quench the reactions. When all time points were quenched, we added 25 μL of 10 mM dithiobisnitrobenzoic acid (DTNB) to the reaction mixture and waited for 5 min to allow the reactions to proceed fully. We then added 1 mL of 0.40 M Tris-Base (pH 8.9) with 20 mM of EDTA. Absorbance of the product 2-nitro-5-thiobenzoic acid (TNB) was immediately measured in a 96 well microplate (Corning, Costar) in a Tecan M1000 Plate Reader at 25 °C. In contrast to an earlier report that TNB was stable in the final solution for at least 2 h at room temperature,[10] we found a small increase in the absorption signal in the final solutions under these conditions, possibly due to alkaline hydrolysis of DTNB at a rate of 0.2% per hour at room temperature and pH 8, forming TNB.[11] Therefore, we measured the absorbance of the final solutions immediately. We used a molar absorption coefficient of 14150 M^{-1} cm^{-1} at 412 nm for TNB.[12] Finally, a DTT consumption rate was calculated based on the measured DTT concentration at different time intervals. DTT loss rate for blank filters was about 0.14 ± 0.06 μM/min. The positive control consisted of 0.5 μM $Cu(II)SO_4$ produced a blank corrected DTT loss rate of 0.75 ± 0.05 μM/min. The average blank corrected DTT signal for samples was about 0.61 μM/min, larger than three times of the standard deviation of blanks by a factor of 3.5.

S1.6 PMF Source Apportionment Analysis

PMF is based on the chemical mass balance equation:

$$x_{ij} = \sum_{k=1}^{p} g_{ik} f_{kj} + e_{ij} \qquad (S5)$$

Where x_{ij} refers to a speciated data set with i samples and j number of species; p refers to the number of factors; g_{ik} is the contribution of the kth factor to ith sample; f_{kj} is the loading of jth species in the kth factor; and e_{ij} represents the residual error for the ith sample and jth species.

PMF solves equation (S5) by minimizing the objective function (Q) (equation (S6)) to derive non-negative factor profiles and contributions:

$$Q = \sum_{i=1}^{n} \sum_{j=1}^{m} (\frac{e_{ij}}{u_{ij}})^2 \qquad (S6)$$

Where n and m refer to the number of samples and species; and u_{ij} is the measurement uncertainty associated with the ith sample and jth species, respectively.[13, 14]

In this study, the OP^{OH}, OP^{DTT} and BC experimental uncertainties were assumed to be three times the standard deviation of the blank measurements, corresponding to a confidence interval of 99.7%. Subsequently, the estimated experimental uncertainty was converted into uncertainty for the volume-normalized OP^{OH}, OP^{DTT} and BC concentrations using the general laws of uncertainty.[15] Uncertainties for the elements were derived by propagating the three major sources of analytical uncertainty: (i) SF-ICPMS measurement; (ii) method blank; and (iii) digestion uncertainty.

Lastly, base model error estimation methods were applied to evaluate the rotational ambiguity and random errors of selected PMF runs. The base model displacement (DISP) analysis requires the decrease in PMF-resolved Q to be < 1% along with no factor swaps for the smallest dQmax = 4.

Additionally, to be considered valid, PMF runs were required to have at least 80% of the factors mapped in the Bootstrap (BS) analysis.[13, 16]

Section S2. Relationships between $PM_{2.5}$ Mass Concentration and Mass-normalized OP^{OH} and OP^{DTT}

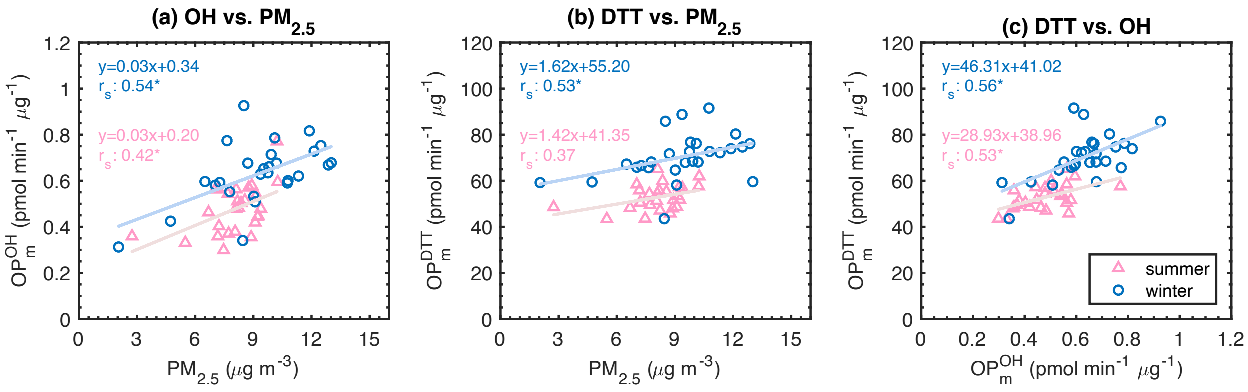

Figure S2. Relationships between OP_m^{OH} and OP_m^{DTT} with $PM_{2.5}$ mass and each other. $p < 0.05$.

Section S3. Correlations between OP and BC, Elements

To understand how OP depends on different chemical components, we calculated Spearman's correlations (r_s) for both volume-normalized and mass-normalized OP^{OH} and OP^{DTT} with BC and 52 elements. For the volume- normalized data, significant correlations were observed between OP and most of the elements, many of which likely resulted from strong correlations between the element and $PM_{2.5}$ mass concentration, making the data difficult to interpret. Mass-normalized OP^{OH} and OP^{DTT} correlations with measured elements were not as strong. Figure S3 shows Spearman's correlations between OP_m^{OH}, OP_m^{DTT} and the elements that were most strongly correlated ($r_s > 0.6$) as well as some selected tracers for dust, biomass burning and sea salt. OP_m^{OH}

was mostly strongly correlated with Zr, Ba and Cu ($r_s = 0.79 – 0.81$), metals associated with brake wear.[17] Ba and Zr were also among the top three elements showing the largest correlations with OP_m^{DTT} ($r_s = 0.72 – 0.74$). The high correlations between OP and brake wear tracers indicate the underlying toxicity of non-exhaust emissions. OP_m^{OH} and OP_m^{DTT} also exhibited moderate to high correlations ($r_s = 0.51 – 0.79$) with tailpipe emission tracers such as BC, Rh, Pd and Pt,[18, 19] in line with many previous studies.[20-22] OP was also correlated with metals associated with dust resuspension, such as Pb and Hg,[23] and metals originating from industrial sources, such as Cd, Ag, and Hf.[24-26] Elements associated with marine emissions including Na, Mg and V[27, 28] overall exhibited negative correlations with OP, which attests to a smaller toxicity of marine aerosols and a dilution effect on OP_m^{OH} and OP_m^{DTT}. Both OP metrics had negative correlations with S, which was largely associated with marine emissions as well as secondary aerosols.

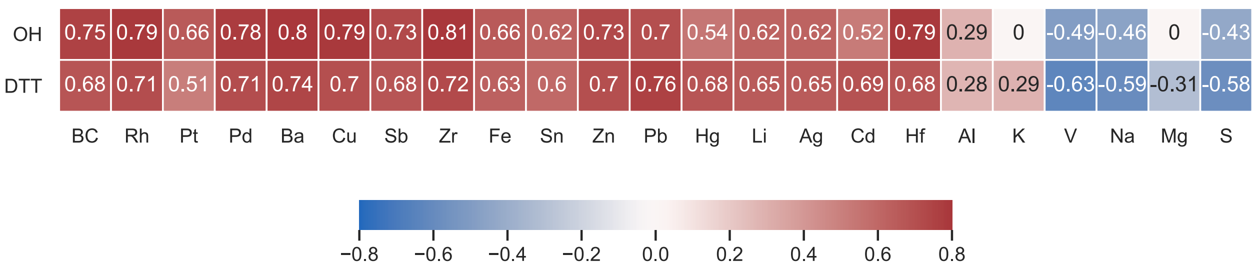

Figure S3. Correlation heatmap showing Spearman's r values ($p < 0.05$) for mass-normalized OP and selected elements.

Section S4. Source Apportionment Results

Uncertainty associated with our OP_v^{OH} and OP_v^{DTT} was evaluated using the Displacement (DISP) and Bootstrap (BS) error estimation tools in PMF. The DISP explores the rotational ambiguity in a PMF solution by assessing the largest range of source profile values without an appreciable increase in the Q-value.[13] For both OP_v^{OH} and OP_v^{DTT} PMF models, the decrease in Q was small

(dQ < 0.01% and < 1% for OP_v^{OH} and OP_v^{DTT}, respectively) and there was no factor swap present for the smallest dQ_{max} ($dQ_{max} = 4$), suggesting there was negligible rotational ambiguity on OP_v^{OH} PMF solution and small rotational ambiguity on OP_v^{DTT} solution. In the BS error estimate, we performed 100 BS runs.

There was no unmapped factor for either the OP_v^{OH} or OP_v^{DTT} PMF solutions. In the OP_v^{OH} PMF solution, 100% of the BS profile was mapped to the base profile for brake and tire wear, mixed secondary and marine and soil and road dust and 81% was mapped for vehicular exhaust source. In the OP_v^{DTT} PMF model, the mapping of BS factors to base profiles ranged from 87% of vehicular exhaust and road dust to 100% of mixed secondary and marine aerosols. Mapping over 80% of all factors indicates random errors were relatively small in our PMF models. In addition, we obtained relatively high R^2 values between the predicted and measured target variables (0.92 and 0.78 for OP_v^{OH} and OP_v^{DTT}, respectively) as shown in Figure S4 and S7. Altogether, this indicates our PMF models are relatively stable and have acceptable statistical characteristics.

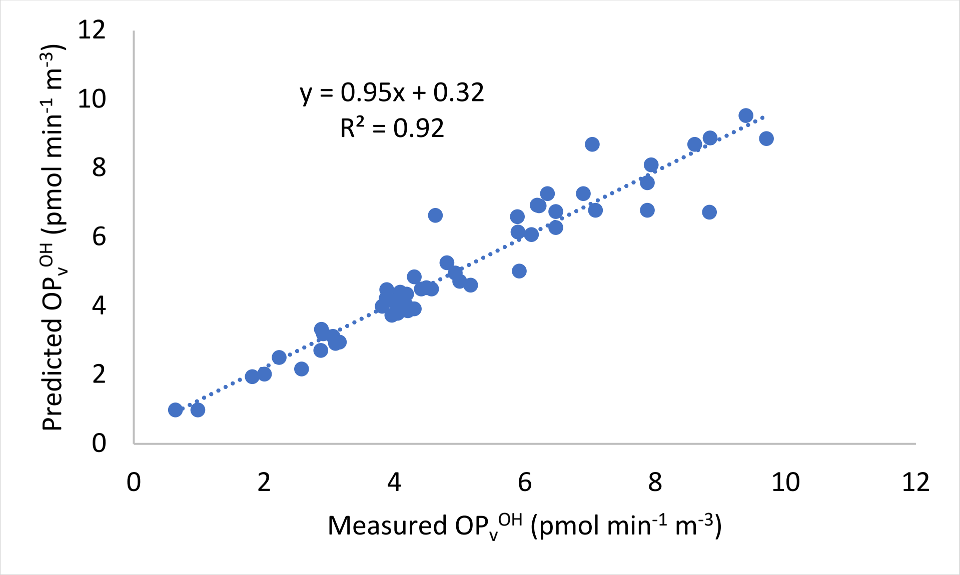

Figure S4. PMF predicted vs. measured OP_v^{OH} for 54 samples.

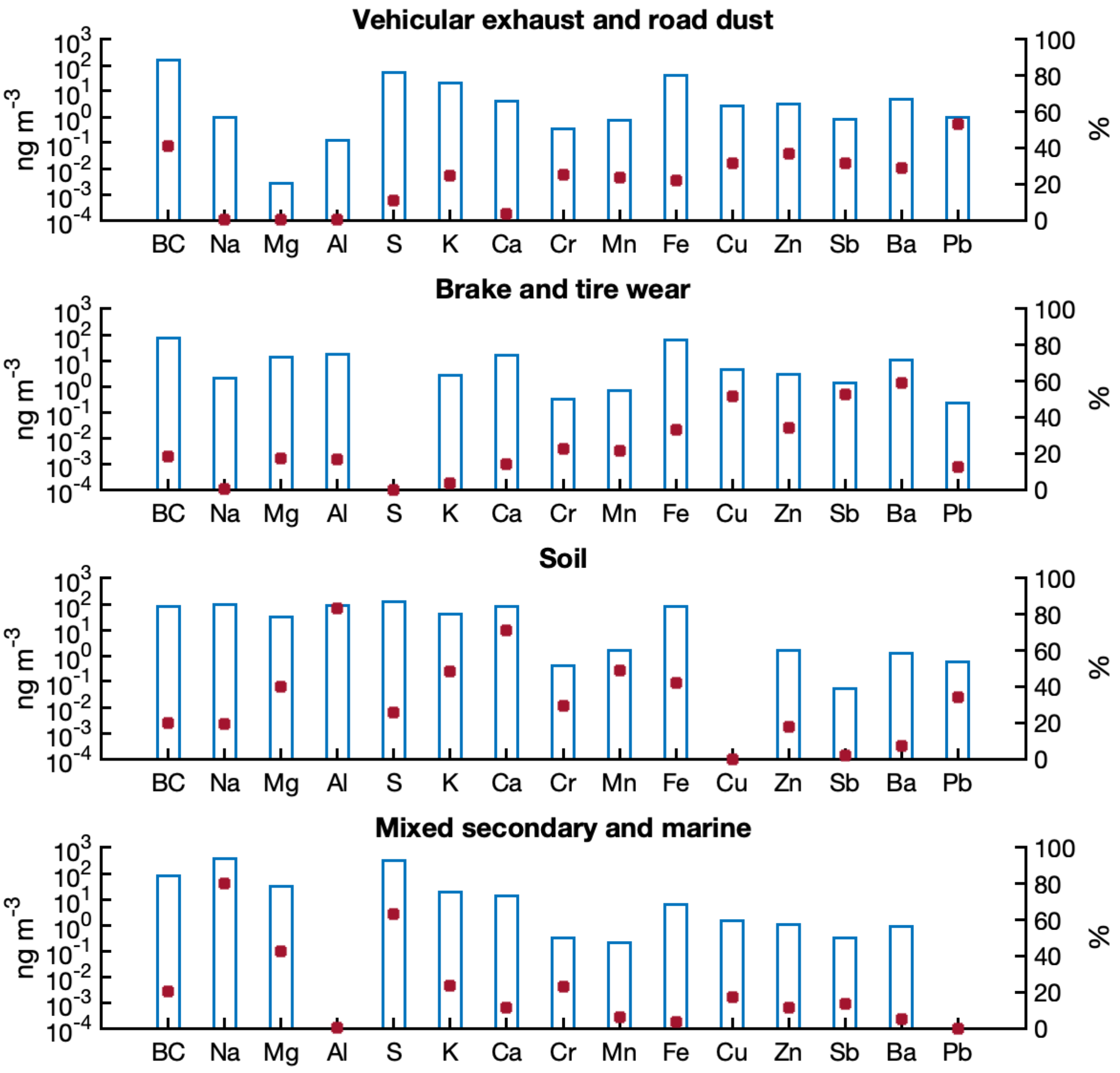

Figure S5. Factor profiles of the OP_v^{DTT} PMF model. The bars (left axis) represent the concentration of species for each factor on a log scale and the dots (right axis) denote the percentage contribution of each factor to the total concentration of each species.

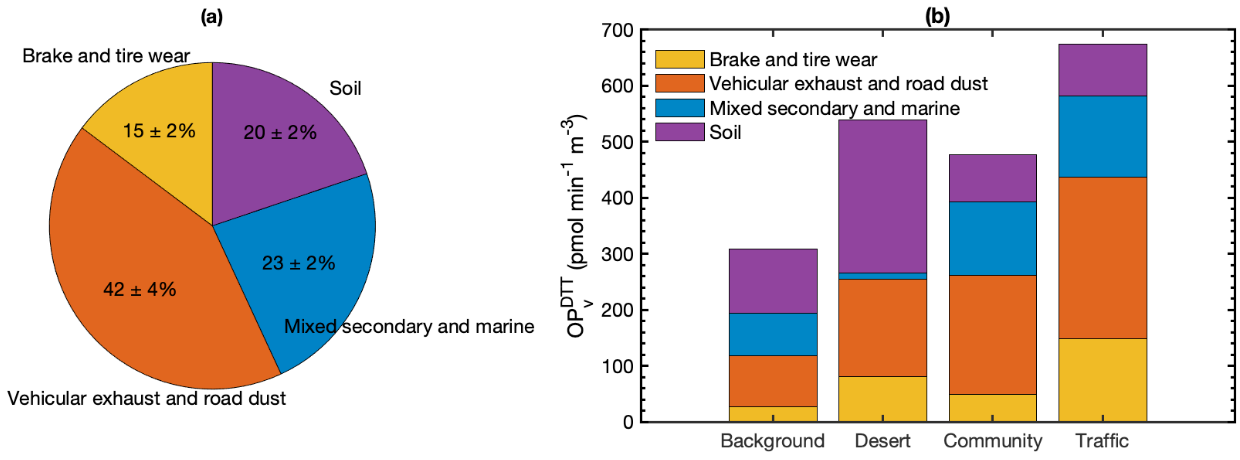

Figure S6. (a) The average contribution of PMF-resolved sources to the OP$_v^{DTT}$ for all sites (both seasons included) with standard error of the mean and (b) for each site category.

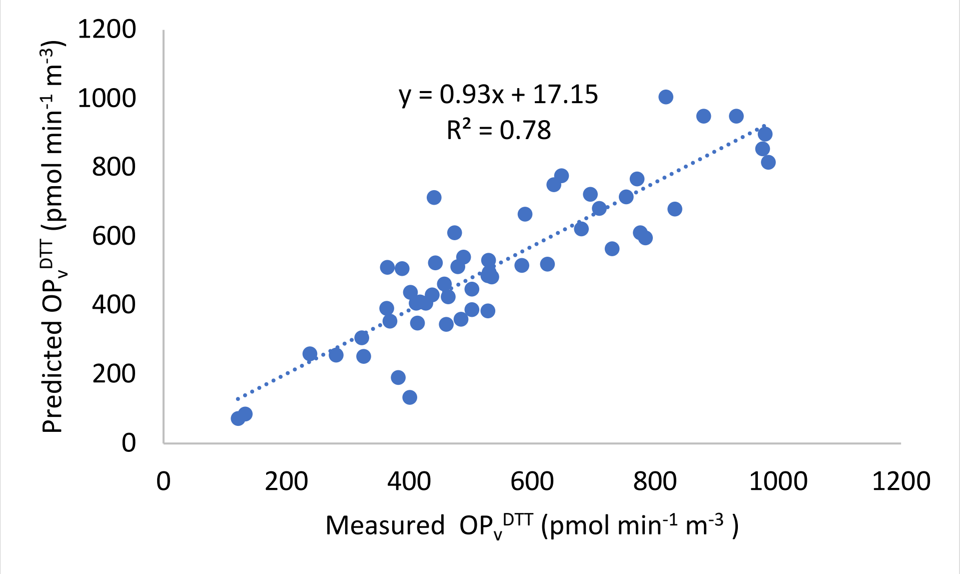

Figure S7. PMF predicted vs. measured OP$_v^{DTT}$ for 54 samples.

Section S5. Oxidative Potential, Pollution Burden and Socioeconomic Position

Table S1. Spearman's correlations for PM2.5 mass, OP and socioeconomic factors.

		This study					CalEnviroScreen socioeconomic indicators				
		$PM_{2.5}$	OP_v^{OH}	OP_v^{DTT}	OP_m^{OH}	OP_m^{DTT}	Educational attainment	Linguistic isolation	Poverty	Unemployment	Housing burden
CalEnviroScreen socioeconomic indicators	Educational attainment	0.30*	0.41*	0.37*	0.47*	0.34*	--				
	Linguistic isolation	0.35*	0.47*	0.41*	0.51*	0.32*	0.75*	--			
	Poverty	0.40*	0.53*	0.44*	0.55*	0.33*	0.87*	0.73*	--		
	Unemployment	0.52*	0.47*	0.36*	0.33*	0.08	0.42*	0.30*	0.56*	--	
	Housing burden	0.41*	0.54*	0.44*	0.53*	0.35*	0.64*	0.59*	0.76*	0.38*	--

* Indicates $p < 0.05$. Numbers without asterisks are not statistically significant at $p < 0.05$. Spearman's r (r_s) > 0.6 are highlighted in green, r_s 0.4 – 0.6 are highlighted in blue, and r_s 0.2 – 0.4 are highlighted in purple.

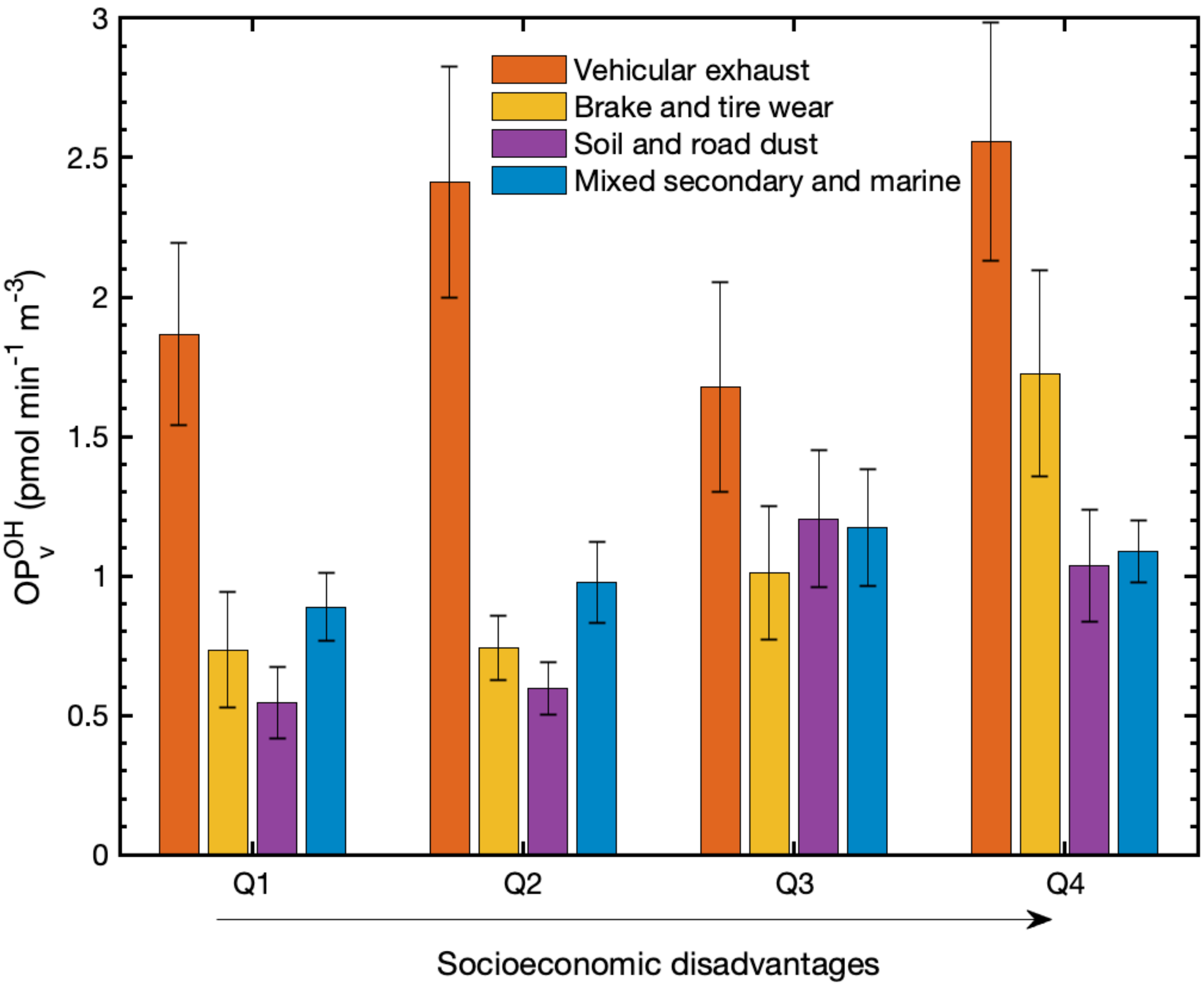

Figure S8. Contribution of four emission sources to the OP_v^{OH} for different socioeconomic position groups.

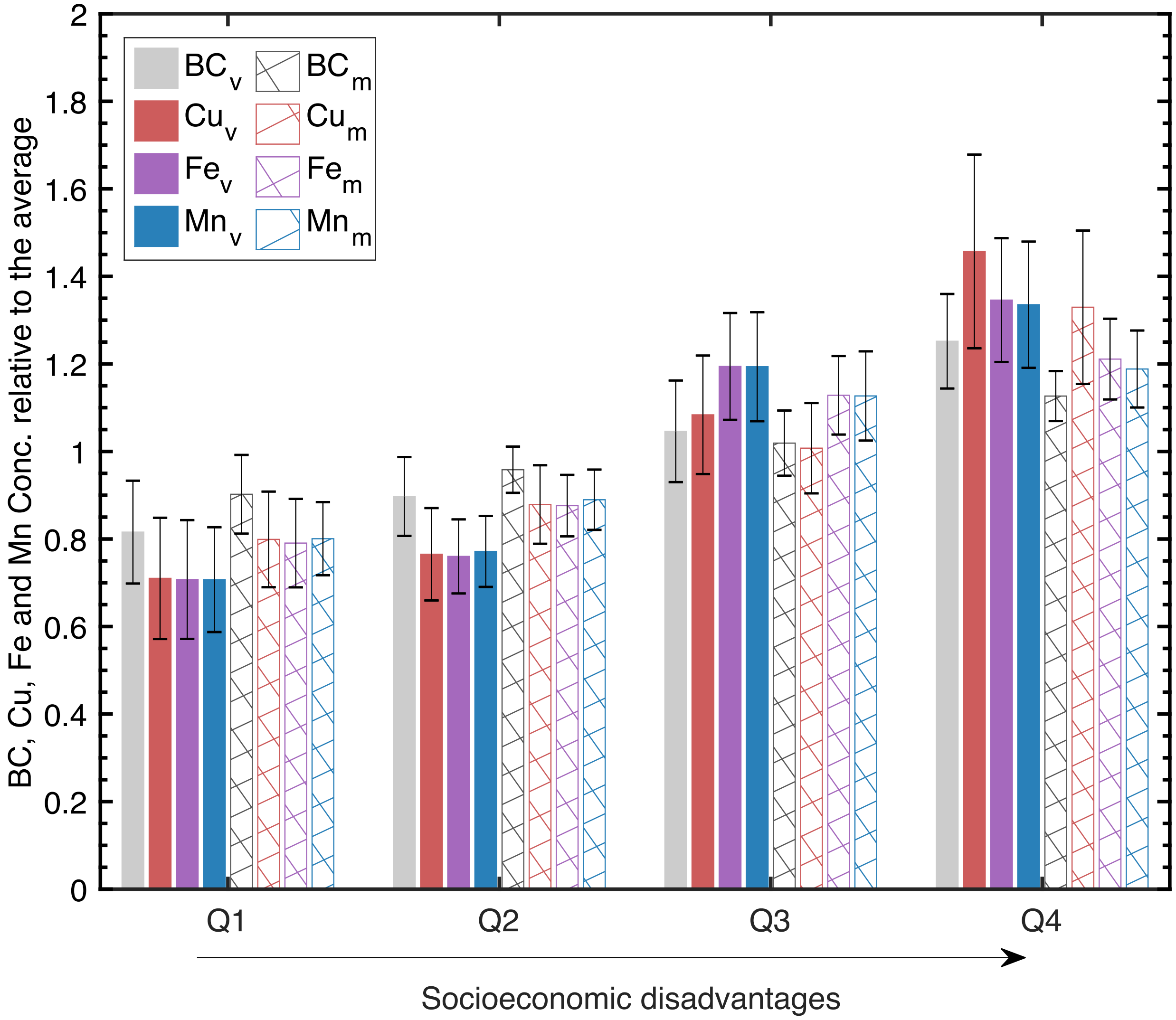

Figure S9. BC, Cu, Fe and Mn relative to their average concentration for each quartile of socioeconomic classification. BC_v, Cu_v, Fe_v and Mn_v denote the volume-normalized element concentration and BC_m, Cu_m, Fe_m and Mn_m refer to the mass-normalized concentration.

Table S2 shows the bivariate Spearman's correlations between $PM_{2.5}$ mass/OP and pollution burden indicators from CalEnviroScreen 4.0. $PM_{2.5}$ mass, OP_v^{OH}, OP_v^{DTT}, OP_m^{OH} and OP_m^{DTT} all exhibited weak to moderate positive correlations with CalEnviroScreen $PM_{2.5}$ concentrations, diesel particulate matter emissions and children's lead risk from housing. Even our $PM_{2.5}$ had a correlation coefficient of only 0.46 with $PM_{2.5}$ in CalEnviroScreen.

Table S2. Spearman's correlations between $PM_{2.5}$ mass or OP and exposure indicators from CalEnviroScreen for the 51 census tracts sampled.

		This study					CalEnviroScreen exposure indicators					
		$PM_{2.5}$	OP_v^{OH}	OP_v^{DTT}	OP_m^{OH}	OP_m^{DTT}	Ozone	$PM_{2.5}$	Diesel PM	Toxic releases	Traffic impacts	Children's lead risk from housing
CalEnviroScreen exposure indicators	Ozone	-0.27*	-0.10	-0.12	0.14	0.14	--					
	$PM_{2.5}$	0.46*	0.52*	0.49*	0.48*	0.34*	-0.14	--				
	Diesel PM	0.48*	0.56*	0.51*	0.50*	0.37*	-0.25	0.61*	--			
	Toxic releases	0.41*	0.32*	0.30*	0.18	0.00	-0.60*	0.50*	0.39*	--		
	Traffic impacts	0.25	0.34*	0.36*	0.30*	0.36*	-0.24	0.28*	0.64*	0.06	--	
	Children's lead risk from housing	0.44*	0.50*	0.44*	0.46*	0.28*	-0.30*	0.47*	0.52*	0.50*	0.21	--

* Indicates $p < 0.05$. Numbers without asterisks are not statistically significant at $p < 0.05$. $r_s > 0.6$ are highlighted in green, r_s 0.4 – 0.6 are highlighted in blue, and r_s 0.2 – 0.4 are highlighted in purple.

References

1. Oroumiyeh, F.; Jerrett, M.; Del Rosario, I.; Lipsitt, J.; Liu, J.; Paulson, S.E.; Ritz, B.; Schauer, J.J.; Shafer, M.M.; Shen, J.; Weichenthal, S.; Banerjee, S.; Zhu, Y., Elemental composition of fine and coarse particles across the greater Los Angeles area: Spatial variation and contributing sources. *Environ. Pollut.* **2022**, 292: p. 118356.
2. Weingartner, E.; Saathoff, H.; Schnaiter, M.; Streit, N.; Bitnar, B.; Baltensperger, U., Absorption of light by soot particles: determination of the absorption coefficient by means of aethalometers. *J. Aerosol Sci.* **2003**, 34(10): p. 1445-1463.
3. Ajtai, T.; Filep, Á.; Utry, N.; Schnaiter, M.; Linke, C.; Bozóki, Z.; Szabó, G.; Leisner, T., Inter-comparison of optical absorption coefficients of atmospheric aerosols determined by a multi-wavelength photoacoustic spectrometer and an Aethalometer under sub-urban wintry conditions. *J. Aerosol Sci.* **2011**, 42(12): p. 859-866.
4. Zotter, P.; Herich, H.; Gysel, M.; El-Haddad, I.; Zhang, Y.; Močnik, G.; Hüglin, C.; Baltensperger, U.; Szidat, S.; Prévôt, A.S., Evaluation of the absorption Ångström exponents for traffic and wood burning in the Aethalometer-based source apportionment using radiocarbon measurements of ambient aerosol. *Atmos. Chem. Phys.* **2017**, 17(6): p. 4229-4249.
5. Vecchi, R.; Bernardoni, V.; Paganelli, C.; Valli, G., A filter-based light-absorption measurement with polar photometer: Effects of sampling artefacts from organic carbon. *J. Aerosol Sci.* **2014**, 70: p. 15-25.

6. Gali, N.K.; Stevanovic, S.; Brown, R.A.; Ristovski, Z.; Ning, Z., Role of semi-volatile particulate matter in gas-particle partitioning leading to change in oxidative potential. *Environ. Pollut.* **2021**, 270: p. 116061.
7. Lack, D.; Cappa, C., Impact of brown and clear carbon on light absorption enhancement, single scatter albedo and absorption wavelength dependence of black carbon. *Atmos. Chem. Phys.* **2010**, 10(9): p. 4207-4220.
8. Gonzalez, D.H.; Kuang, X.M.; Scott, J.A.; Rocha, G.O.; Paulson, S.E., Terephthalate probe for hydroxyl radicals: yield of 2-hydroxyterephthalic acid and transition metal interference. *Analytical Lett.* **2018**, 51(15): p. 2488-2497.
9. Cho, A.K.; Sioutas, C.; Miguel, A.H.; Kumagai, Y.; Schmitz, D.A.; Singh, M.; Eiguren-Fernandez, A.; Froines, J.R., Redox activity of airborne particulate matter at different sites in the Los Angeles Basin. *Environ. Res.* **2005**, 99(1): p. 40-47.
10. Charrier, J.G.; Anastasio, C., On dithiothreitol (DTT) as a measure of oxidative potential for ambient particles: evidence for the importance of soluble transition metals. *Atmos. Chem. Phys. (Print)* **2012**, 12(5): p. 11317-11350.
11. Riddles, P.W.; Blakeley, R.L.; Zerner, B., Ellman's reagent: 5, 5′-dithiobis (2-nitrobenzoic acid)—a reexamination. *Anal. Biochem.* **1979**, 94(1): p. 75-81.
12. Eyer, P.; Worek, F.; Kiderlen, D.; Sinko, G.; Stuglin, A.; Simeon-Rudolf, V.; Reiner, E., Molar absorption coefficients for the reduced Ellman reagent: reassessment. *Anal. Biochem.* **2003**, 312(2): p. 224-227.
13. Norris, G.; Duvall, R.; Brown, S.; Bai, S., EPA positive matrix factorization (PMF) 5.0 fundamentals and user guide. **2014**: p. 1-136.
14. Reff, A.; Eberly, S.I.; Bhave, P.V., Receptor modeling of ambient particulate matter data using positive matrix factorization: review of existing methods. *J. Air Waste Manage. Assoc.* **2007**, 57(2): p. 146-154.
15. Farrance, I.; Frenkel, R., Uncertainty of measurement: a review of the rules for calculating uncertainty components through functional relationships. *Clin. Biochem. Rev.* **2012**, 33(2): p. 49.
16. Brown, S.G.; Eberly, S.; Paatero, P.; Norris, G.A., Methods for estimating uncertainty in PMF solutions: examples with ambient air and water quality data and guidance on reporting PMF results. *Sci. Total Environ.* **2015**, 518: p. 626-635.
17. Adachi, K.; Tainosho, Y., Characterization of heavy metal particles embedded in tire dust. *Environ. Int.* **2004**, 30(8): p. 1009-1017.
18. McDonald, B.C.; Goldstein, A.H.; Harley, R.A., Long-term trends in California mobile source emissions and ambient concentrations of black carbon and organic aerosol. *Environ. Sci. Technol.* **2015**, 49(8): p. 5178-5188.
19. Lough, G.C.; Schauer, J.J.; Park, J.-S.; Shafer, M.M.; DeMinter, J.T.; Weinstein, J.P., Emissions of metals associated with motor vehicle roadways. *Environ. Sci. Technol.* **2005**, 39(3): p. 826-836.
20. Lovett, C.; Sowlat, M.H.; Saliba, N.A.; Shihadeh, A.L.; Sioutas, C., Oxidative potential of ambient particulate matter in Beirut during Saharan and Arabian dust events. *Atmos. Environ.* **2018**, 188: p. 34-42.

21. Shirmohammadi, F.; Hasheminassab, S.; Wang, D.; Schauer, J.J.; Shafer, M.M.; Delfino, R.J.; Sioutas, C., The relative importance of tailpipe and non-tailpipe emissions on the oxidative potential of ambient particles in Los Angeles, CA. *Faraday Discuss.* **2016**, 189: p. 361-380.
22. Cheung, K.; Ntziachristos, L.; Tzamkiozis, T.; Schauer, J.; Samaras, Z.; Moore, K.; Sioutas, C., Emissions of particulate trace elements, metals and organic species from gasoline, diesel, and biodiesel passenger vehicles and their relation to oxidative potential. *Aerosol Sci. Technol.* **2010**, 44(7): p. 500-513.
23. Najmeddin, A.; Keshavarzi, B.; Moore, F.; Lahijanzadeh, A., Source apportionment and health risk assessment of potentially toxic elements in road dust from urban industrial areas of Ahvaz megacity, Iran. *Environ. Geochem. Health* **2018**, 40(4): p. 1187-1208.
24. Viana, M.; Pandolfi, M.; Minguillón, M.; Querol, X.; Alastuey, A.; Monfort, E.; Celades, I., Inter-comparison of receptor models for PM source apportionment: case study in an industrial area. *Atmos. Environ.* **2008**, 42(16): p. 3820-3832.
25. Taghvaee, S.; Sowlat, M.H.; Mousavi, A.; Hassanvand, M.S.; Yunesian, M.; Naddafi, K.; Sioutas, C., Source apportionment of ambient PM2. 5 in two locations in central Tehran using the Positive Matrix Factorization (PMF) model. *Sci. Total Environ.* **2018**, 628: p. 672-686.
26. Chang, Y.; Huang, K.; Xie, M.; Deng, C.; Zou, Z.; Liu, S.; Zhang, Y., First long-term and near real-time measurement of trace elements in China's urban atmosphere: temporal variability, source apportionment and precipitation effect. *Atmos. Chem. Phys.* **2018**, 18(16): p. 11793-11812.
27. Adachi, K.; Buseck, P.R., Changes in shape and composition of sea-salt particles upon aging in an urban atmosphere. *Atmos. Environ.* **2015**, 100: p. 1-9.
28. Corbin, J.C.; Mensah, A.A.; Pieber, S.M.; Orasche, J.; Michalke, B.; Zanatta, M.; Czech, H.; Massabò, D.; Buatier de Mongeot, F.; Mennucci, C., Trace metals in soot and PM2. 5 from heavy-fuel-oil combustion in a marine engine. *Environ. Sci. Technol.* **2018**, 52(11): p. 6714-6722.
